# Aortic atheroma as a source of stroke – assessment of embolization risk using 3D CMR in stroke patients and controls

**DOI:** 10.1186/s12968-017-0379-x

**Published:** 2017-09-06

**Authors:** Thomas Wehrum, Iulius Dragonu, Christoph Strecker, Florian Schuchardt, Anja Hennemuth, Johann Drexl, Thomas Reinhard, Daniel Böhringer, Werner Vach, Jürgen Hennig, Andreas Harloff

**Affiliations:** 1 Department of Neurology, Medical Center - University of Freiburg, Breisacher Straße 64, 79106 Freiburg, Germany; 2 Faculty of Medicine, University of Freiburg, Freiburg, Germany; 3 Fraunhofer MEVIS, Bremen, Germany; 4 Eye Center, Medical Center, University of Freiburg, Freiburg, Germany; 5 Institute for Medical Biometry and Statistics, University of Freiburg, Freiburg, Germany; 6 Department of Diagnostic Radiology – Medical Physics, Medical Center, University of Freiburg, Freiburg, Germany

**Keywords:** Atherosclerosis, Aorta, Stroke, Multi-contrast MRI, 4D flow MRI

## Abstract

**Background:**

It was our purpose to identify vulnerable plaques in the thoracic aorta using 3D multi-contrast CMR and estimate the risk of cerebral embolization using 4D flow CMR in cryptogenic stroke patients and controls.

**Methods:**

One hundred patients (40 with cryptogenic stroke, 60 ophthalmologic controls matched for age, sex and presence of hypertension) underwent a novel 3D multi-contrast (T1w, T2w, PDw) CMR protocol at 3 Tesla for plaque detection and characterization within the thoracic aorta, which was combined with 4D flow CMR for mapping potential embolization pathways. Plaque morphology was assessed in consensus reading by two investigators and classified according to the modified American-Heart-Association (AHA) classification of atherosclerotic plaques.

**Results:**

In the thoracic aorta, plaques <4 mm thickness were found in a similar number of stroke patients and controls [23 (57.5%) versus 33 (55.0%); *p* = 0.81]. However, plaques ≥4 mm were more frequent in stroke patients [22 (55.0%) versus 10 (16.7%); *p* < 0.001]. Of those patients with plaques ≥4 mm, seven (17.5%) stroke patients and two (3.3%) controls (*p* < 0.001) had potentially vulnerable AHA type VI plaques. Six stroke patients with vulnerable AHA t﻿ype VI plaques ≥4 mm had potential embolization pathways connecting the plaque, located in the aortic arch (*n* = 3) and proximal descending aorta (*n* = 3), with the individual territory of stroke, which made them the most likely source of stroke in those patients.

**Conclusions:**

Our findings underline the significance of ≥4 mm thick and vulnerable plaques in the aortic arch and descending aorta as a relevant etiology of stroke.

**Clinical trial registration:**

Unique identifier: DRKS00006234; date of registration: 11/06/2014

**Electronic supplementary material:**

The online version of this article (doi:10.1186/s12968-017-0379-x) contains supplementary material, which is available to authorized users.

## Background

Atherosclerosis of the aorta is regarded as a source of embolic stroke, if complex plaques (≥4 mm thick, ulcerated, or containing mobile thrombi) are detected in the ascending aorta and the aortic arch by transesophageal echocardiography (TEE) [1]. In clinical practice, the search for aortic plaques is usually initiated when stroke etiology remains cryptogenic after routine diagnostics. Interestingly, more than 50% of patients with cryptogenic stroke show atheroma <4 mm in the ascending aorta and proximal aortic arch and/or plaques ≥4 mm in the distal arch and descending aorta [2]. Research investigating carotid and coronary arteries has shown that not only plaque size but also plaque composition influences plaque vulnerability and thus the risk of rupture and subsequent vascular events. A thin cap with a large lipid core, endothelial denudation, fissures, superficial calcifications, and intraplaque hemorrhage are criteria of vulnerable plaques [3] which can only in part be assessed by TEE. Furthermore, plaques of the distal arch (i.e. the proximal descending aorta) which are located downstream of the left subclavian artery were identified as a potential source of stroke in patients with otherwise cryptogenic stroke etiology [4]. Their embolic potential arises from the physiological end diastolic retrograde blood flow which is able to transport plaque material back into the proximal aortic arch and thus ultimately to the brain- supplying arteries [4–6]. TEE is neither able to accurately locate plaques with respect to the branches of the supraaortic vessels [7] nor to visualize potential embolization pathways [8]. Therefore, it was our aim to detect atheroma (particularly ≥4 mm thick and vulnerable thoracic plaques) among patients with cryptogenic ischemic stroke and ophthalmologic controls using a novel 3D multi-contrast CMR protocol [9]. Furthermore, in order to investigate if embolization pathways to the brain-supplying arteries originating from such atheroma are more frequent in stroke patients and to assess the role of this mechanism for ischemic stroke, we visualized embolization pathways [10] with 4D flow CMR.

## Methods

### Study cohort

#### Stroke patients

Inclusion criteria for stroke patients were: a) age ≥ 50 years, b) acute brain ischemia with visible acute lesion on brain MRI, and c) cryptogenic stroke etiology after routine diagnostics including TEE. Between September 2014 and March 2015, 745 patients ≥50 years of age were admitted to our hospital for symptoms consistent with ischemic stroke and were screened for eligibility for this study within 24–48 h after admission. All patients underwent routine diagnostics (i.e. brain CT, MRI or both, Doppler ultrasound of extra- and intracranial arteries, transthoracic- and transesophageal echocardiography, 12-lead and Holter-ECG, routine laboratory tests and additionally factor V Leiden mutation, antithrombin III, protein C and S, and antiphospholipid antibody titer in patients <60 years of age).

One hundred sixty nine patients had no visible brain lesion on diffusion weighted MRI and were thus excluded from the study, leaving 576 patients to be further evaluated. Of those, the stroke etiology following modified TOAST criteria was determined in 406 patients (70.5%): large-artery atherosclerosis in 195 (33.8%), cardioembolism in 141 (24.5%), small-vessel disease in 38 (6.6%) and other causes in 32 (5.6%) patients. In 170 (29.5%) patients stroke etiology remained cryptogenic despite complete and thorough diagnostic workup. We included only cryptogenic stroke patients in order to systematically evaluate the prevalence of plaques of the aortic arch and descending aorta and of potential embolization pathways that constitute a likely source of cerebral embolism. Of these 170 patients, 19 had CMR-contraindications, 23 were not legally competent to give consent due to severe stroke/aphasia, 50 required intensive care, 17 declined study participation, 10 were already transferred to another hospital or discharged before study CMR could be conducted, 8 were severely obese (BMI ≥35) and thus not suitable for our CMR protocol. Accordingly, 43 patients were scheduled for a study CMR. One patient aborted the examination and two had to be excluded because atrial fibrillation was detected after performance of study CMR (=determined stroke etiology). Thus, complete CMR data of 40 acute stroke patients were available for final analysis.

Cardiovascular risk factors and National Institutes of Health Stroke Scale (NIHSS) were prospectively documented in all participants based on personal interviews and patients’ medical charts.

#### Controls

Patients from the Eye Center without a history of retinal or cerebral ischemia as indicated by personal interview and patients’ medical charts were chosen as controls. Based on data from a previous study (prevalence of complex plaques: 46% in cryptogenic stroke patients and 19% in controls) [6] we calculated sample size to be *n* = 60 in the control group with a power of 80%, alpha error of 0.05, and an enrollment ratio of 1.5. Accordingly, 60 controls were frequency matched to stroke patients in terms of age, sex, and hypertension and underwent CMR. Cardiovascular risk factors were prospectively documented in all participants based on personal interviews and patients’ medical charts.

The study was approved by the University of Freiburg Ethics Committee and informed consent was obtained from all participants.

### Aortic CMR

All CMR examinations were executed on a 3 Tesla system (PRISMA, Siemens Healthcare AG, Erlangen, Germany) using a 12-element body coil. All sequences were ECG-synchronized and respiration-controlled using navigator-gating (T1w, 4D flow) and triggering (T2w, PDw), respectively.

#### Plaque imaging

The multi-contrast CMR protocol consisted of a T1w bright-blood (3D GRE, echo time/repetition time (TE/TR) = 1.62 ms/6.1 ms, flip-angle = 12°, acceleration = GRAPPA (*R* = 2, 32 ref. lines), a T2w black-blood (3D SPACE, TE/TR = 103 ms/average duration respiratory cycle, flip-angle = 90°, acceleration = elliptical scanning, partial Fourier 6/8), and a PDw black-blood (3D SPACE, TE/TR = 23 ms/average duration respiratory cycle, flip-angle = 90°, acceleration = elliptical scanning, partial Fourier 6/8) sequence. All multi-contrast sequences had the same geometric parameters: Field-of-view (FOV) = 264 × 240 mm^2^, matrix = 256 × 178, 56 partitions resulting in a nearly-isotropic spatial resolution of 1.15 × 1.15 × 1.17 mm^3^. T1w bright-blood images of the aorta were acquired using a RF spoiled GRE sequence with 41 segments per cardiac cycle. For fat suppression, a binomial 1–2-1 water excitation pulse was used. T2w and PDw dark-blood images of the aorta were acquired using a 3D RARE sequence with variable non-selective refocusing flip angles (Sampling Perfection with Application optimized Contrasts by using different flip angle Evolutions: SPACE; Siemens Healthcare) [11]. Efficient luminal signal suppression was obtained using double inversion recovery (DIR) pulse [12] in combination with 3D SPACE which was used because of its inherent dark-blood capabilities.

#### Flow imaging

Furthermore, 4D flow CMR was used to visualize 3D blood flow within the thoracic aorta. Parameters were: TE/TR = 2.6 ms/5.1 ms, flip angle = 7°, temporal resolution = 40 ms, matrix = 320x240x58, bandwidth = 450 Hz/pixel, spatial resolution =2.1 × 2.1 × 2.5mm^3^, velocity sensitivity along all three directions = 150 cm/s, and parallel imaging (PEAK-GRAPPA) along the phase encoding direction (y) with an acceleration factor of *R* = 5 (20 reference lines).

### Detection of aortic plaques

All datasets were pseudonymized and analyzed in consensus reading by two investigators with experience in CMR-based plaque analysis of five and 8 years, who were blinded to patient data and affiliation to the case or control group. In the case of a disagreement among observers, a third investigator with experience in CMR-based plaque analysis of 10 years made the decision. To analyze intra- and inter-rater variability and to avoid recognition of datasets, one observer repeated the analysis 3 months and 6 months after baseline and was blinded to results of the previous analysis. Plaques within the thoracic aorta were detected based on 3D multi-contrast CMR. The aorta was subdivided into the ascending aorta (AAo, area between the aortic valve and the outlet of the brachiocephalic trunc (BcT)), the aortic arch (AA, area between the outlet of the BcT and the left subclavian artery (LSA) comprising all parts of the vessel wall at equal level as the branching sites), and the descending aorta (DAo; area distal to the outlet of the LSA). Three-dimensional T1w, T2w, and PDw CMR images were displayed using multiplanar reformatting and synchronization to allow immediate assessment of all contrasts at one time. The DAo was scrutinized for plaques as far as 6 cm distal to the LSA.

#### Assessment of image quality

Image quality was rated separately for each sequence as good (=2: vessel wall can be clearly distinguished in all parts of the thoracic aorta), satisfactory (=1: occurrence of blurring and flow artefacts in parts of the thoracic aorta but image quality still sufficient for reliable assessment), and bad (=0: not evaluable; i.e. motion artefacts, blurring and insufficient suppression of blood signal leading to an inability to reliably assess the circumference of the aortic wall).

#### Vulnerable plaques

**Fig. 1:**
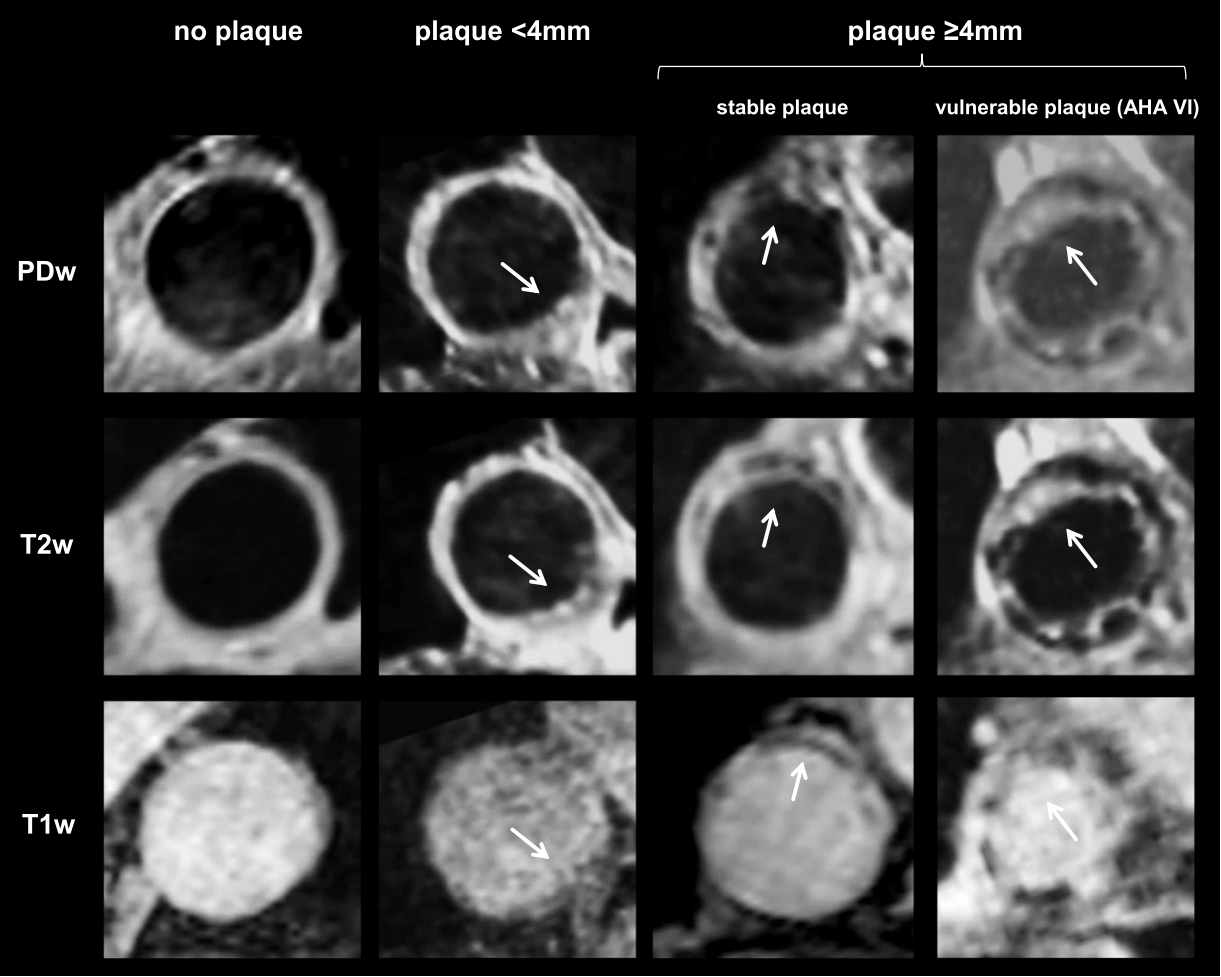
Examples of multi-contrast plaque imaging (T1w, T2w, PDw) CMR images. The first column shows a segment of the aortic arch without a plaque, the second shows a plaque <4 mm-thickness, the third shows a stable plaque and the fourth shows a vulnerable plaque ≥4 mm-thickness (AHA type VII plaque with a heterogeneous composition with iso- and hyperintense lipid-rich areas, hypointense nodular calcifications and a rough plaque surface with ulcerations)

Plaque thickness (i.e. distance between lumen-intima and media-adventitia interface) was quantified by manual measurements of axial reconstructions of T2w 3D images of the aorta using electronic calipers at the site of the plaques largest diameter in cross-section. Plaque composition was evaluated regarding features determining plaque vulnerability (surface irregularities, superimposed thrombus, T1-hyperintensities indicating fresh hemorrhage, calcification, etc.) and classified according to the modified AHA classification for CMR (see Table 1 and Fig. 1) [13]. AHA type VI plaques were regarded as potentially vulnerable plaques. The intensity of the cardiac muscle served as reference to grade signal intensities as iso-, hypo- and hyperintense. The appearance of plaque components was defined as follows [13, 14]: Lipid deposits were defined as iso- to hyperintense regions within the plaque on both T1w and PDw images and as hypointense regions on T2w images. Calcium components were defined as hypointense regions within the plaque on T1w, PDw, and T2w images. Fibrotic components were defined as isointense regions of the plaque on T1w, PDw, and T2w images. Fresh intraplaque hemorrhage was defined as a hyperintense signal on T1w and an isointense signal of T2w and PDw images whereas recent hemorrhage is identified by a hyperintense signal on all three contrasts.

**Table 1:** Modified AHA classification of atherosclerotic plaque

Modified AHA Classification for aortic CMR (derived from carotid MRI [13])	
Type I–II: near-normal wall thickness, no calcification, not visible because of limited spatial resolution	
Type III: intimal thickening with no calcification or small eccentric plaque without calcification	
Type IV–V: plaque with a lipid or necrotic core surrounded by fibrous tissue with possible calcification	
Type VI: complex plaque with possible surface defect, hemorrhage, or thrombus	
Type VII: calcified plaque without features of VI	
Type VIII: fibrotic plaque without lipid core and with possible small calcifications without features of VI

### Visualization of potential embolization pathways

**Fig. 2:**
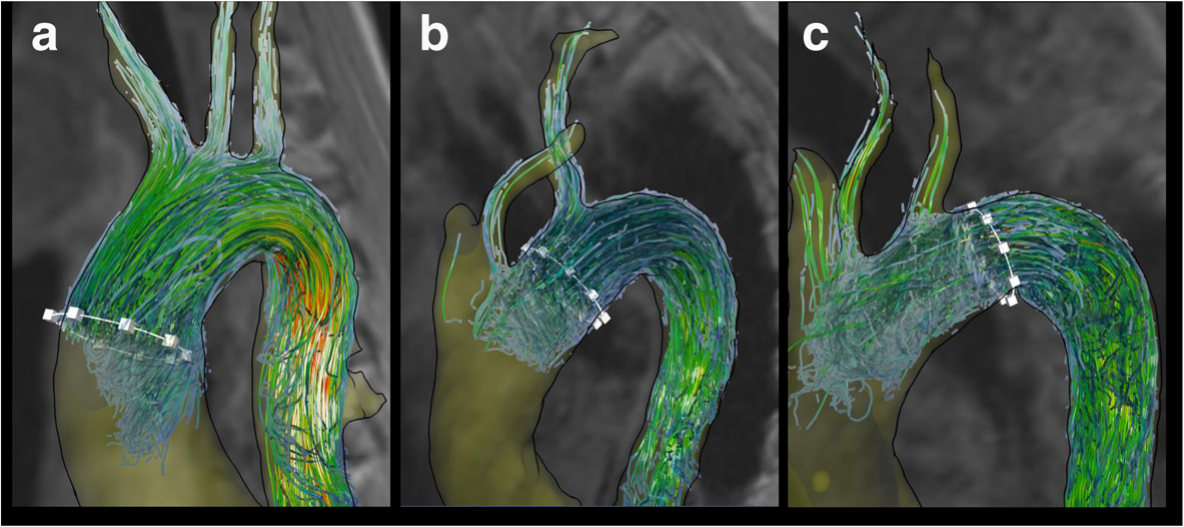
Examples of embolization pathway mapping using 4D flow CMR. **a** A plaque located in the ascending aorta (white region of interest (ROI)) has potential embolization pathways to all brain territories. **b** A plaque in the aortic arch (white ROI) has potential antegrade embolization pathways via the LSA and retrograde pathways via the LCCA. **c** A plaque in the distal arch (i.e. proximal descending aorta; white ROI) shows potential retrograde embolization pathways via the BcT, LCCA and LSA

4D flow CMR datasets were processed and analyzed using MEVISFlow software (Fraunhofer MEVIS, Bremen, Germany). The processing workflow consisted of correction of velocity offset errors and velocity aliasing artifacts, vessel segmentation and particle tracking within time-resolved and three-dimensional flow data (for technical details see [10]). Two-dimensional planes of aortic plaques ≥4 mm were imported from multi-contrast CMR and were co-registered with 4D flow CMR data in each patient. An emitter plane was positioned at the site of the plaque and time-resolved three-dimensional particle traces resembling blood flow originating at this site were generated [6]. A possible embolization pathway was defined as present in case of co-occurrence of a vulnerable plaque and blood flow connecting this plaque with the outlet of a brain-supplying artery. Possible embolization was defined as present in stroke patients with a combination of aortic plaques and 3D blood flow pathways reaching the outlet of a) the LSA, b) the left common carotid artery (LCCA), and c) the brachiocephalic trunk (BCT) if the vessel reached by retrograde flow trajectories supplied the brain territory affected by brain ischemia on cerebral MRI (see Fig. 2 and Additional file 1: Video 1).

### Statistical analysis

Data are presented as mean (±standard deviations) or median (interquartile range) for continuous, absolute and relative frequencies for categorical variables. Departures from normality were detected with the Shapiro-Wilk statistic. Differences between patient groups were evaluated using chi-square tests and Fisher’s exact tests for qualitative and independent samples, t-tests for quantitative parameters. A Mood’s median test was used to test if median plaque prevalence was identical in cases and controls. The weighted Cohen’s kappa (κ) coefficient was used to quantify inter- and intra-rater agreement as excellent (κ ≥ 0.75), fair to good (κ0.40 to 0.75), and poor (κ < 0.40). All tests were two-sided with 0.05 as the level of statistical significance. Statistical analyses were performed using IBM-SPSS Statistics version 19.0.1.

## Results

### Baseline characteristics

Demographics and cardiovascular risk factors of all study participants are provided in Table 2. Median stroke severity according to the NIHSS was 2 (interquartile range = 0–4). All stroke patients underwent brain MRI with diffusion weighted imaging after admittance. Seven (17.5%) patients had a right-sided, 14 (35.0%) had a left-sided ischemic lesion, 16 (40.0%) had ischemic defects in the posterior circulation, and 3 (7.5%) had lesions in multiple vascular territories.

**Table 2:** Patients’ demographics and cardiovascular risk factors

Characteristics of patients	Stroke patients *n* = 40	Controls *n* = 60	*p*-value
Age, years (±SD)	68.7 (±9.7)	68.2 (±9.1)	0.78
Female, *n* (%)	27 (67.5)	39 (65.0)	0.80
Hypertension, *n* (%)	26 (65.0)	43 (71.7)	0.48
Hyperlipidemia, *n* (%)	20 (50.0)	21 (35.0)	0.14
Diabetes, *n* (%)	8 (20.0)	7 (11.7)	0.25
Smokers, *n* (%)	13 (32.5)	21 (35.0)	0.80
BMI, 1 (±SD)	27.1 (4.2)	26.5 (3.7)	0.44
Previous stroke/TIA, *n* (%)	12 (30.0)	0 (0.0)	-
Coronary heart disease, *n* (%)	6 (15.0)	8 (35.0)	0.81
Peripheral arterial disease, *n* (%)	2 (5.0)	1 (1.7)	0.34
Mean systolic BP, mmHg (±SD)	140.0 (±15.7)	138.2 (±13.8)	0.54
Mean diastolic BP, mmHg (±SD)	76.8 (±9.8)	79.2 (±9.3)	0.22
Heart rate, bpm (±SD)	64.2 (±10.3)	64.9 (±10.7)	0.74

*TIA* transient ischemic attack, *BP* blood pressure *SD* standard deviation

### Results from CMR aortic plaque imaging

Duration of the complete protocol after placing the patient inside the scanner was 59:25 ± 9:27 min (7:29 ± 2:10 min for localizers, 8:49 ± 2:47 min for T1w, 14:06 ± 5:46 min for T2w, 12:00 ± 3:23 min for PDw, and 17:01 ± 3:05 min for 4D flow CMR). Image quality was rated good in 97% and satisfactory in 3% for T1w, good in 93% and satisfactory in 7% for T2w, and good in 80% and satisfactory in 20% for PDw images. Accordingly, all sequences were assessable. Results of intra- and inter-rater agreement of AHA plaque classification were as follows: inter-rater reliability was ĸ = 0.83 (*p* < 0.001, 95% CI 0.67, 0.98) and intra-rater variability was ĸ = 0.96 (*p* < 0.001, 95% CI 0.87, 1.0).

### Plaque prevalence in the thoracic aorta

#### On a patient level

Twenty-three (57.5%) stroke patients and 33 (55.0%) controls (*p* = 0.81) had plaques of any degree (<4 mm and ≥4 mm) in the thoracic aorta. Every subject (i.e. 23 stroke patients and 33 controls) with aortic plaques had at least one small plaque (<4 mm). The median number of plaques <4 mm was 2 (range: 1–5) in stroke patients and 1 (range: 1–3) in controls (*p* = 0.02). Large plaques (≥4 mm) were found in 22 (55.0%) stroke patients and 10 (16.7%) controls (*p* < 0.001). The median number of plaques ≥4 mm was 1 (range: 1–4) in stroke patients and 1 (range: 1–2) in controls (*p* = 0.78).

#### On a group level

A total of 51 plaques <4 mm and 34 plaques ≥4 mm were found in stroke patients. In controls a total of 51 plaques <4 mm and 11 plaques ≥4 mm were found. Plaques <4 mm were located in the following sections of the aorta in stroke patients and controls: 8 and 4 in the ascending aorta, 13 and 12 in the aortic arch, and 30 and 35 in the descending aorta. Plaques ≥4 mm were located: 1 and 0 in the ascending aorta, 7 and 1 in the aortic arch, and 26 and 10 in the descending aorta.

### CMR Plaque classification according to AHA

Irrespective of plaque thickness, we classified 53 and 53 plaques as AHA Type III, 8 and 0 as AHA Type IV-V, 7 and 2 as AHA Type VI (=vulnerable plaques), 12 and 6 as AHA Type VII and 5 and 1 as AHA Type VIII. Accordingly, there were more patients with at least one advanced plaque (AHA type IV-VIII) in the group of stroke patients (20/40 (50.0%)) than in controls (9/60 (15%)) (*p* < 0.001), whilst the frequency of patients with AHA type III plaques was similar (10/40 (33.3%) vs. 26/60 (43.0%); *p* = 0.903). Large plaques (≥4 mm) were potentially vulnerable AHA Type VI plaques in 7/22 (31.8%) and 2/10 (20%) cases (*p* = 0.68).

### Prevalence of embolization pathways

**Fig. 3:**
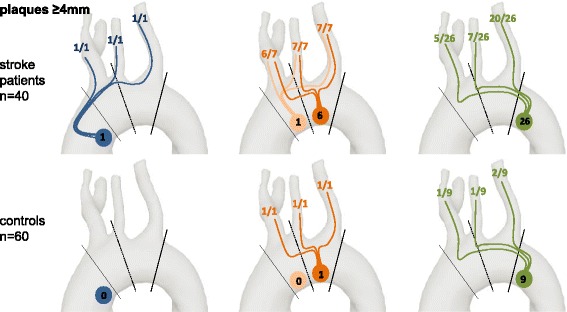
Number of plaques ≥4 mm in each section of the aorta. The total number of plaques ≥4 mm in each section of the aorta (blue: AAo, orange: AA, green: DAo) is given for cases and controls. Potential embolization pathways to the BcT, LCCA, and LSA are displayed for the respective plaque localization/ aortic segment

Embolization pathways connected the site of large plaques ≥4 mm with a supraaortic artery in 20 cases and 3 controls: the BCT in 12 and 2, the LCCA in 15 and 2, and the LSA in 28 and 3 (Fig. 3). The relative likelihood of the presence of potential embolization pathways originating from the site of a plaque ≥4 mm is given in Table 3. Accordingly, a connection between plaques in the AAo (1/1100%), AA (7/7100%), and the DAo (16/26, 61.5%) and the territory of stroke could be established in 18 stroke patients with ischemia of the posterior circulation (*n* = 10), the left (*n* = 4) and right (*n* = 3) hemisphere, and involvement of multiple vascular territories (*n* = 1). Of these, one patient had simultaneous plaques in the AAo, the AA, and the DAo and six had simultaneous plaques in the AA and DAo, which had a connection to the affected stroke territory.

**Table 3:** Relative likelihood of presence of potential embolization pathways originating from plaques ≥4 mm in relation to the location in the aorta

Origin of pathlines		Supraaortic arteries reached by pathlines		
		BCT	LCCA	LSA
AAo	stroke	100%	100%	100%
	controls	-	-	-
AA	stroke	85.7%	100%	100%
	controls	100%	100%	100%
DAo	stroke	19.2%	26.9%	76.9%
	controls	11.1%	11.1%	22.2%

*AAo* ascending aorta, *AA* aortic arch, *DAo* descending aorta, *BCT* brachiocephalic trunc, *LCCA* left common carotid artery, *LSA* left subclavian artery

### The culprit lesion – Matching of embolization pathways with cerebral imaging

**Fig. 4:**
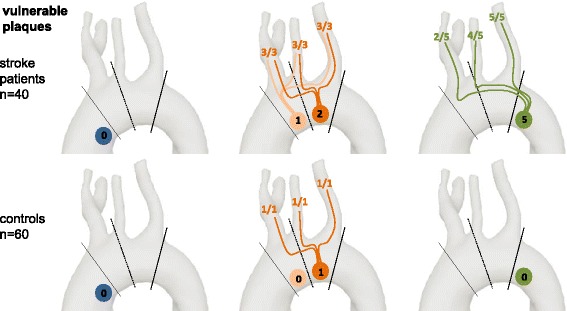
Number of vulnerable AHA type VI plaques in each section of the aorta. Analogously to Fig. 3, the number of vulnerable AHA type VI plaques and potential embolization pathways are displayed for each aortic segment

Of the 18 stroke patients with plaques ≥4 mm and potential embolization pathways to the individual territory of stroke, AHA plaques types were: type III in 1, type IV in 2, type VI in 6, type VII in 5, and type VIII in 4 patients. In patients with vulnerable AHA type VI plaques, those were located in the aortic arch (*n* = 1), the DAo (*n* = 3), and in both the aortic arch and the DAo (*n* = 2). In the absence of a known stroke etiology, those plaques might be defined as culprit plaques, i.e. vulnerable plaques which probably caused stroke. In control patients, we detected only one potential vulnerable plaque in the aortic arch (=type VI) with a possible connection to all brain territories, and two plaques of uncertain vulnerability in the DAo (AHA type VII) with a potential retrograde embolization pathway reaching the LSA and the BCT (Fig. 4).

## Discussion

In the present study, we examined patients with cryptogenic stroke etiology and an acute ischemic lesion in cerebral MRI and compared them with controls regarding the prevalence of aortic plaques. We focused on large and vulnerable plaques of the aortic arch and proximal descending aorta, and potential embolization pathways to the brain. Age-, sex-, and hypertension-matched ophthalmic patients were regarded as optimal controls because they were free of the outcome of interest (i.e. stroke), representative of the population at risk of the outcome, and independent of the exposure of interest. Furthermore, to our knowledge, this is the first study to apply a complete 3D multi-contrast protocol tailored to aortic plaque detection to a large group of patients. The CMR sequences used in this study showed high intra- and inter-observer agreement regarding image quality grading of the 3D T1w, T2w, and PDw sequences [9] and regarding assessment of 4D flow pathlines using the same sequence and analysis software [10]. Furthermore, we report high intra- and inter-rater agreement of plaque classification evaluation according to AHA in this study. Accordingly, we believe that both the 3D multicontrast CMR sequences and the 4D flow sequence used in this study are highly reliable.

No difference was observed between groups regarding the prevalence of small plaques (<4 mm thickness) throughout the aorta, which were found in more than half of all studied patients. However, despite equal frequencies of systemic cardiovascular risk factors, stroke patients had significantly more plaques ≥4 mm thickness in the entire thoracic aorta than controls, which is in accordance to the literature [6], but also a significantly higher frequency of potentially vulnerable AHA type VI plaques. Visualization of embolization pathways indicated that all plaques in the ascending aorta and the proximal aortic arch, but also >60% of plaques in the proximal DAo ≥4 mm thickness had at least one potential connection to a brain-supplying artery.

Within the last years, there has been a controversy regarding the role of plaques in the DAo as a source of cerebral embolism [15, 16]. However, ten patients of our study with otherwise cryptogenic brain infarction had an isolated plaque ≥4 mm in the proximal DAo which was potentially vulnerable in five cases (=50%). Furthermore, all patients had a visible lesion on cerebral MRI and no other apparent cause of stroke despite extended workup including TEE, underlining the causal relationship between complex DAo plaques and brain embolism. In controls, however, we found only one potential vulnerable plaque in the aortic arch (=type VI) with a possible connection to all brain territories and two plaques of uncertain vulnerability in the DAo (AHA type VII) with a potential retrograde embolization pathway reaching the LSA and the BCT. This predominance of large and vulnerable plaques in stroke patients emphasizes the role of such atheroma in the DAo for embolic stroke.

In this study, we used a novel 3-dimensional multi-contrast plaque imaging protocol to identify vulnerable plaques and 4D flow CMR to visualize potential embolization pathways from such plaques in the DAo to the brain. The multi-contrast plaque protocol comprised a T1w bright blood sequence which proved to be reliable for the detection of aortic plaques [17] supplemented by T2w and PDw dark blood sequences. The combined analysis of signal intensities in different sequences allowed to delineate the vessel wall and evaluate the structure of the plaque surface as well as different plaque components such as calcifications (hypointense in T1w, T2w, and PDw), lipid-rich areas (iso- hyperintense in T1w and PDw, hypointense in T2w), hemorrhages (hyperintense in T1w), and fibrosis of different stages (variable signal intensities). Previous multi-contrast CMR protocols were 2-dimensional [18–24]. Thus a) the region of interest had to be selected prior to data acquisition, b) the 3-dimensional geometry of a plaque could not be visualized, and c) subjects had to hold their breath during data acquisition, which excludes severely ill and incompliant patients. As shown in our study, 3D–multi-contrast CMR is able to overcome such restrictions. However, the limits of this technique are the required longer scan times and motion artefacts especially in the ascending aorta and proximal aortic arch.

Multi-contrast plaque CMR has already been used to classify plaques according to the AHA classification in the carotid arteries. However, AHA originally is a histological classification which was extended to CMR for carotid plaque classification after direct comparison with histologic specimen [13]. Only one study performed CMR of the aorta for plaque detection before surgical grafting of abdominal aortic aneurysm and therefore provided comparison of in-vivo CMR with histology [22]. Yet, only the straight, down-stream segment of the aorta was investigated, which most probably is not relevant for stroke. Accordingly, a drawback of our approach using the modified AHA classification is the lack of histology as a reference method as patients did not undergo surgery. Nevertheless, we believe that by using the same contrasts, signal intensities of different types of tissue in the aorta are comparable to those of the carotid arteries where comparisons with histology are possible. Another issue may be overestimation of plaque thickness in CMR, which is due to the fact that the media cannot be clearly differentiated from the adventitia. Those differences may be insignificant, as indicated in a previous study [25].

By adding 4D flow measurements to our protocol we were able to detect potential embolization pathways to the brain. This is especially important for plaques located distal to the outlets of the brain supplying arteries in the proximal descending aorta. However, aortic flow reversal varies between individuals [26] and even large plaques in the descending aorta are found in many patients without stroke. Accordingly, 4D flow CMR is necessary to discriminate if a plaque that is located in the proximal DAo may be the source of a cerebral embolism. Hence, assessment of embolization pathways using 4D flow CMR may guide treatment in those patients as lacking potential embolization pathways may warrant the search for an alternative source of stroke. Currently, the estimation of likelihood of potential embolization pathways is based on visual analysis of pathlines. A more detailed estimation would consider the uncertainty that is based on noise, motion artifacts, and on measurement errors included in the 4D flow CMR data. Such uncertainty calculation is complex but mathematically possible [27]. However, it was not yet available for analysis of our 4D flow data but would be a valuable addition for data analysis in the future.

### Limitations

Ideally, a T1w dark blood sequence would have been implemented in order to better evaluate the vessel wall to further facilitate the discrimination of vessel lumen and wall. Implementation of a 3D T1w dark-blood sequence for the aorta was technically difficult to realize at that time. However, this limitation was compensated by the two additional black-blood contrasts applied during 3D T2 and PD weighted imaging in all patients. Using these three complementary CMR contrasts, we believe that even hyperintense plaque components in T1 weighted CMR were reliably discriminated from the bright blood signal. Multi-contrast plaque imaging protocols in the carotid arteries usually also comprise a time-of-flight (TOF) sequence which is used to detect fresh intra-plaque hemorrhage [26]. However, TOF imaging cannot be used in the aorta because of the large field of view and the resulting expenditure of measurement time. An alternative for better imaging of fibrosis and hemorrhage within the wall of the aorta may be a 3D late gadolinium enhancement sequence in future patients. However, this would have required the application of contrast agents which was ethically not possible in control subjects, as it did not guide treatment in those patients. Furthermore, the duration of our multi-contrast protocol plus 4D flow CMR comprised nearly 1 h and could therefore not be performed on critically ill patients. The long scan time also resulted in a limited sample size due to restricted CMR capacities which required focusing on cryptogenic stroke patients as a subgroup to achieve a sufficiently high power to detect differences in plaque prevalence. Hence, further achievements are necessary to reduce scan time or increase sample size and improve spatial resolution.

## Conclusions

In conclusion, we have shown that our novel multi-contrast CMR protocol is capable to depict plaques in 3D and throughout the entire thoracic aorta. This protocol proved to be robust in 100 patients and should be considered for future trials in order to characterize plaques in analogy to the carotid arteries and identifying vulnerable lesions. In addition, we found that despite matching for age and cardiovascular risk factors, cryptogenic stroke patients more often had complex and vulnerable plaques in the aortic arch and the descending aorta compared to controls and thus a higher frequency of potential embolization pathways.

## Additional file

Additional file 1:
**Video 1.** Potential embolization pathlines of a 4 mm-thick and vulnerable plaque in an acute stroke patient. This 4 mm-thick plaque, classified as AHA Type VI, is located at the level of the outlet of the left subclavian artery in an acute stroke patient. Potential embolization pathlines originating at the site of the plaque are able to reach all brain-supplying arteries including the territory of stroke, which was located in the left hemisphere in this patient. (MP4 603 kb)

## Abbreviations

AA: Aortic arch
AAo: Ascending aorta
AHA: American Heart Association classification
BcT: Brachiocephalic trunc
DAo: Descending aorta
DIR: Double inversion recovery
FOV: Field-of-view
LSA: Left subclavian artery
NIHSS: National Institutes of Health Stroke Scale
SPACE: Sampling perfection with application optimized contrasts by using different flip angle evolutions
TEE: Transesophageal echocardiography
